# Decoupling Colloidal Stability and Catalytic Activity of Gold Nanocatalysts via In-Situ Sacrificial Physisorbed PEG Coatings

**DOI:** 10.1021/acs.langmuir.6c01965

**Published:** 2026-06-02

**Authors:** Andrew A. Pettenger, Shayd Gothard, Tuhina Banerjee, Santimukul Santra

**Affiliations:** Department of Chemistry and Biochemistry, 7471 Missouri State University, 901 S. National Avenue, Springfield, Missouri 65897, United States

## Abstract

Gold nanoparticles stabilized by sodium citrate (GNPs–citrate) are highly effective catalysts but often suffer from colloidal instability. Polymeric stabilizers can improve dispersion stability, yet chemisorbed polymer layers are canonically associated with suppressed catalytic activity. Herein, we evaluate an in situ sacrificial stabilizing system (ISSS) based on *physisorbed* hydroxyl-terminated poly­(ethylene glycol) (PEG) on GNPs–citrate. GNPs–PEG were prepared using PEG (1.5, 4.0, and 6.0 kDa) at Au/PEG molar ratios of 1:2, 1:7, and 1:12. Dynamic light scattering showed substantially increased hydrodynamic diameter (*D*_h_) and decreased magnitude of ζ-potential with increasing PEG loading, while UV–visible spectroscopy indicated invariant surface plasmon resonance (λ_SPR_) features relative to GNPs–citrate. In the NaBH_4_ reduction of *p*-nitrophenol to *p*-aminophenol, GNPs–PEG exhibited pseudo-first-order rate constants with no statistically significant difference to those of GNPs–citrate across the series; at 8.0 μM Au, the mean activity differed from the citrate control by only ∼3%, and no induction period was observed. Prolonged storage preserved *D*_h_, ζ-potential, and λ_SPR_, whereas stability tests under reaction conditions showed declining activity and increasing *D*_h_ over time, consistent with the expected loss of the physisorbed PEG layer. Collectively, these data support physisorbed PEG as a storage-stabilizing coating that is labile under reducing catalytic conditions, enabling stability without any sacrifice to initial catalytic performance.

## Graphical abstract

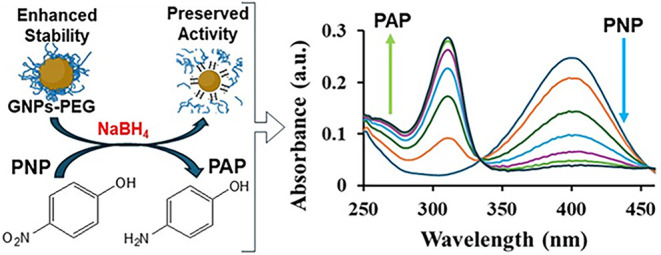

## Introduction

Gold nanoparticles (GNPs) are small clusters of gold (Au) atoms, typically defined within the range of 1–100 nm, and can exist as colloidal dispersions in aqueous media or grafted to a variety of porous, solid support materials. Interest in GNPs has grown substantially due to their potential applications in theranostics, optics, environmental management, and catalysis.^1^ GNPs and other metallic NPs are popular subjects of study due to the unique properties that emerge from their nanostructures. For example, and most pertinent to the research presented here, GNPs are associated with characteristically high surface-area-to-volume ratios, precisely what makes them such effective catalysts.^2,3^

The synthesis of GNPs has been studied extensively for decades, with numerous methods developed to control particle size, shape, and surface chemistry.^4–7^ Much more recently, many impressively novel synthetic routes have been developed, each offering unique circumstantial advantages.^8–13^ Among the various approaches, however, chemical reduction of gold salts in solution remains the most widely used, owing to its simplicity, scalability, and ability to produce well-defined colloids. In particular, the citrate reduction method introduced by Turkevich and later refined by Frens has become a cornerstone of nanoparticle synthesis.^7^ In this process, tetrachloroauric acid (HAuCl_4_) is reduced by citrate at elevated temperatures. Citrate simultaneously serves as a reducing agent and an electrostatic stabilizer, forming GNPs with citrate-stabilizing coats (GNPs-citrate). By adjusting the gold-to-citrate ratio, particles with diameters spanning roughly 9–120 nm can be reproducibly obtained, with smaller ratios favoring the formation of smaller, more monodisperse particles. Variants of the method employ UV irradiation or ascorbic acid as reductants at room temperature, yielding similar products with subtle differences in shape and dispersion. Beyond citrate-based routes, a range of other reductants and stabilizers, including amino acids, surfactants, and radiation chemistry, have been explored to tailor particle morphology.^7^ This variety of techniques demonstrates the rich tunability of GNP properties through methodically designed reaction conditions.

GNPs have been established as versatile catalysts across a broad range of transformations in both gas- and liquid-phase systems. In heterogeneous catalysis, Au-based materials are widely discussed in the context of low-temperature CO oxidation and other reactions where activity depends strongly on particle size and the Au–support interface.^14^ In liquid-phase catalysis, polymer-stabilized Au nanoclusters have been shown to promote aerobic alcohol oxidation in water, illustrating pronounced size-dependent activity even among colloidal Au catalysts.^15^ GNPs are also reported to catalyze chemoselective hydrogenation reactions (e.g., nitroaromatic hydrogenation) under supported-catalyst conditions, again with activity and selectivity tied to Au size and support effects.^16^ Beyond “classic” redox chemistry, GNPs have been reported as catalysts for C–C bond-forming cross-couplings such as Suzuki–Miyaura reactions in aqueous media, expanding their relevance to synthetic methodology.^17^ More recently, Au nanostructures have become central in electrocatalysis, including CO_2_-to-CO conversion, where selectivity can vary with GNP size and shape.^18^ Because GNP catalytic performance is frequently controlled by particle size, Au–support contact structure, and the chemical nature/dynamics of surface stabilization, these structure–interface effects are emphasized in modern reviews of Au catalysis.^19^ Relatedly, several studies have shown that AuNP catalyst performance and recyclability can be strongly influenced by stimulus-responsive surface chemistry that enables reversible dispersion/phase transfer and catalyst recovery, providing practical routes to control catalyst accessibility and reuse in liquid-phase systems.^20,21^ Finally, the NaBH_4_ reduction of *p*-nitrophenol is commonly used as a convenient aqueous benchmark for probing how reaction conditions and surface chemistry influence catalytic induction times and observed reaction rates.^22^ The catalytic mechanism in this reaction is most commonly described in the literature using the Langmuir–Hinshelwood model.^23^ The extensive range of chemical transformations that can be catalyzed by GNPs via numerous catalytic methods justifies the broad interest in these nanostructures and underscores the need for research that optimizes their utility and further extends the reach of their applicability.

One of the most significant obstacles commonly encountered when working with GNPs is their high tendency to aggregate. It is well documented that the colloidal stability of GNP dispersions in aqueous media will collapse over time, resulting in aggregation and the consequential loss of the unique properties that make them valuable in the first place.^24–26^ This challenge has motivated extensive research aimed at improving colloidal stability and minimizing aggregation. For colloidal dispersions of GNPs, the most popular method of enhancing stability is through surface functionalization with polymeric stabilizing systems. These stabilizing systems are commonly composed of polymeric ligands that form strong bonding interactions with the Au surface. These chemically adsorbed polymer layers are known, however, to attenuate catalytic performance through surface passivation, diffusion barriers, and the emergence of an induction period.^27^ Colloidal GNPs, therefore, exist on a stability-to-catalytic-activity trade-off spectrum, and this research was intended to provide novel positioning on this spectrum. In contrast to applications like theranostics, in which biocompatibility is essential and thus maximized stability is an obvious objective, the role of stability in catalytic applications is less obvious and should be evaluated on a case-by-case basis. For catalytic applications, “optimal” stability must balance colloidal persistence against catalytic surface accessibility.

In addition to this trade-off spectrum, recent results published by Neal et al.^28^ and Ansar et al.^29^ help to further establish precedent for this research. These studies showed that even strongly bound organothiols will desorb completely and spontaneously over time in NaBH_4_. This is attributed to the more stable bond formed between hydride and the Au surface, which effectively displaces the thiolated ligands. Together, these results suggest that chemically bound ligand desorption can be slow enough for surface passivation and diffusion barriers to weaken initial catalytic performance and produce induction periods. This problem has been previously addressed in the literature by suggesting methods for the proactive removal of ligands from the Au surface, prior to use in catalysis. These methods, however, often involve grafting the GNPs to a solid support material.^27,30^

Finally, it has been reported that neutral, hydroxylated poly­(ethylene) glycol (PEG) forms physically adsorbed (physisorbed) coatings around GNPs-citrate.^31,32^ In the absence of specialized terminal groups, physisorbed coatings are formed via polyvalent supramolecular interactions. The study by Xue et al.,^31^ in particular, serves as a foundational work that investigates important properties regarding the formation of these systems. The study found that PEG physically adsorbs to the GNPs via electrostatic interactions with citrate on the metallic surface, rather than displacing the citrate and adsorbing to the Au surface directly. In contrast to thiolated polymers, there is no thermodynamic incentive for a standard ligand exchange to occur. Furthermore, the study determined that the adsorption of PEG to citrate-covered GNPs occurs primarily through hydrogen bonding and that the PEG chains adopt a loop-and-train-tail conformation, with increasing molecular weights resulting in larger loops and longer tails. This study also investigated the binding efficiency and strength of physisorbed PEG on GNPs-citrate. Using isothermal titration calorimetry (ITC), the authors reported an impressively large equilibrium constant and thermodynamically favorable enthalpy of formation for the adsorption of 20 kDa PEG: *K*_a_ ≈ 1.06 × 10^6^ and Δ*H* ≈ −198.1 kcal/mol. Additionally, for this same system, the binding stoichiometry was measured as ∼8.7 PEG chains/GNP. This was relatively consistent with their NMR-based estimate of ∼10.0 PEG chains/GNP. The authors also reported a clear inverse relationship between the quantified surface density and the molecular weight of physisorbed PEG: 0.132, 0.052, 0.034, and 0.020 σ/nm^2^ for 2.0, 6.0, 10.0, and 20.0 kDa PEG, respectively. These results establish PEG physisorption on GNPs-citrate as a significant interfacial interaction worthy of further study across broader applications.

Physisorption of PEG on GNPs-citrate is, however, rarely studied relative to its chemically adsorbed counterparts and, to the best of our knowledge, has yet to be extensively studied in the context of catalysis. Based on the foundational concepts previously described, we herein report a proof-of-principle that demonstrates the feasibility of an in situ sacrificial stabilizing system (ISSS), which takes advantage of the electrostatic interactions that are characteristic of physisorption and are weaker than chemisorbed counterparts. GNPs-citrate with increasingly large, physisorbed PEG coatings (GNPs-PEG) were prepared and shown to incur no statistically significant loss in initial catalytic performance for the reduction of *p*-nitrophenol (PNP) to *p*-aminophenol (PAP) using sodium borohydride (NaBH_4_), a popular model reduction reaction for evaluating the kinetics of catalytic metals.^33,34^ Stability assays in the PNP reduction medium indicated efficient desorption of the physisorbed PEG layers, a plausible explanation for the preserved activity. Furthermore, long-term storage tests showed excellent preservation of colloidal and dimensional properties.

## Results and Discussion

### Synthesis and Characterization of GNPs-PEG

GNPs-citrate were prepared by the standard citrate reduction of HAuCl_4_. They were then mixed with linear PEG*_n_* (*n* = 1.5, 4.0, and 6.0 kDa) at defined Au/PEG molar ratios to allow noncovalent adsorption, yielding a series of GNPs-PEG*_n_* (Au/PEG). Scheme 1 summarizes GNPs-PEG synthesis, their application in PNP reduction, and the proposed desorption mechanism. The total mass of PEG used in each preparation of GNPs-PEG*_n_* (Au/PEG) is provided in Table 1. No thiols or coupling reagents were used; the PEG layer was therefore expected to be physisorbed rather than chemisorbed. This decoupled core formation from interfacial modification, keeping the metallic core constant while varying the PEG coating thickness. The following characterizations verify these assumptions and quantify how physisorbed PEG alters the hydrodynamic and interfacial environment. The results of dynamic light scattering (DLS) characterization revealed a clear trend in both hydrodynamic diameter (*D*_h_) and surface charge (ζ-potential) properties across the GNPs-PEG series. Representative *D*_h_ and ζ-potential plots are provided in Figure 1.

**Figure 1:**
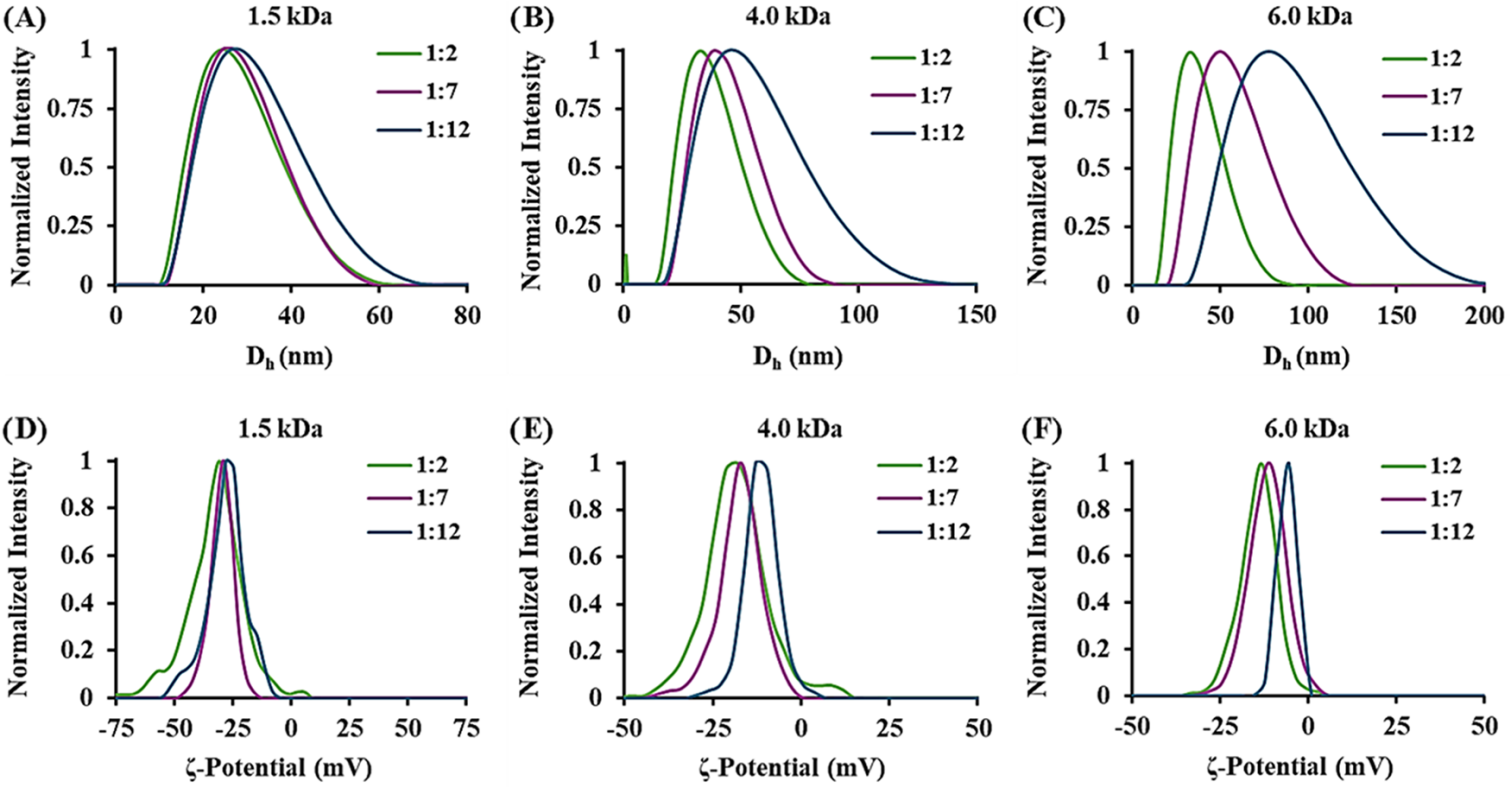
Representative plots of *D*_h_ (A–C) and ζ-potential (D–F) for GNPs-PEG using 1.5 (A, D), 4.0 (B, E), and 6.0 (C, F) kDa of PEG.

**Scheme 1:**
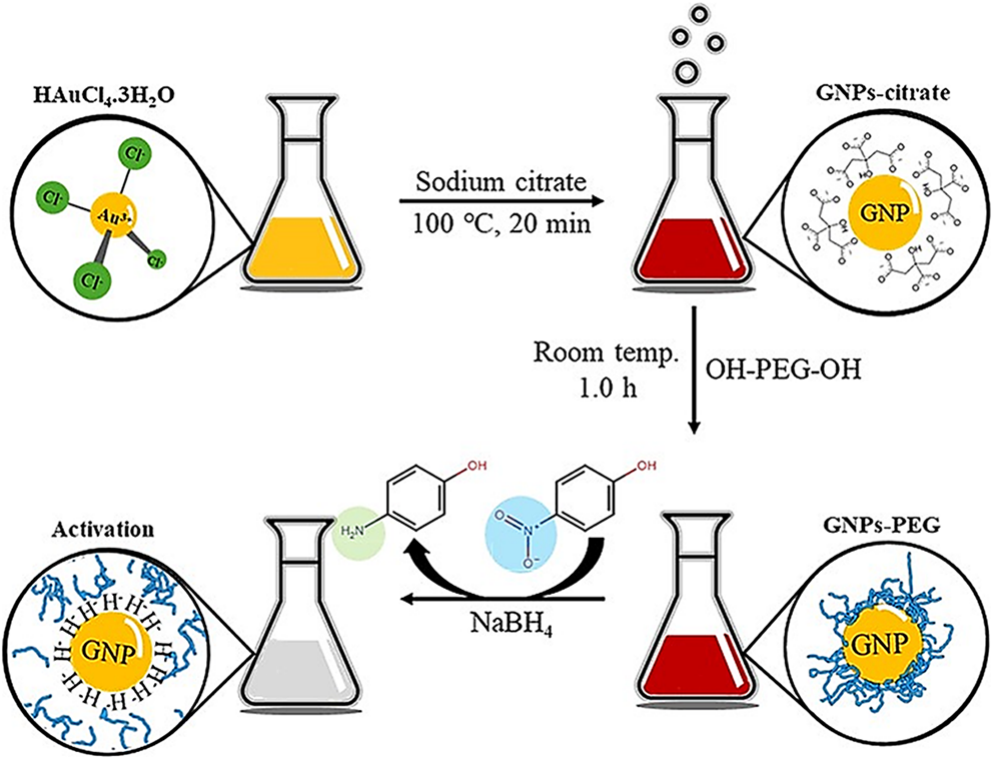
Summary of GNPs-PEG Synthesis and Application in the Catalytic Reduction of PNP to PAP using NaBH_4_

**Table 1:** Total Mass of PEG in Each Preparation of GNPs-PEG

preparation Number	1	2	3	4	5	6	7	8	9
PEG M.W. (kDa)	1.5	1.5	1.5	4.0	4.0	4.0	6.0	6.0	6.0
Au/PEG (mol)	1:2	1:7	1:12	1:2	1:7	1:12	1:2	1:7	1:12
total PEG Mass (mg)	150	525	900	400	1400	2400	600	2100	3600

The average experimental values of *D*_h_ and ζ-potential are tabulated in the Supporting Information (SI, Table S1) and are consistent with the successful formation of physisorbed polymer coatings around GNPs-citrate. As seen in Figure 2A, the *D*_h_ of GNPs-PEG increased proportionally with increasing total mass of PEG, and the ζ-potential became less negative as total mass of PEG and *D*_h_ increased (Figure 2B). Furthermore, a relationship approximating that of an exponential decay in the magnitude of the ζ-potential as *D*_h_ increased was observed. This trend is shown in Figure 2C,D, and the corresponding linearized transformation supports an approximate exponential fit (*R* ^2^ = 0.94; standard error of regression = 0.003). Corresponding characterization values for GNPs-citrate are indicated by the red dot in Figure 2.

**Figure 2:**
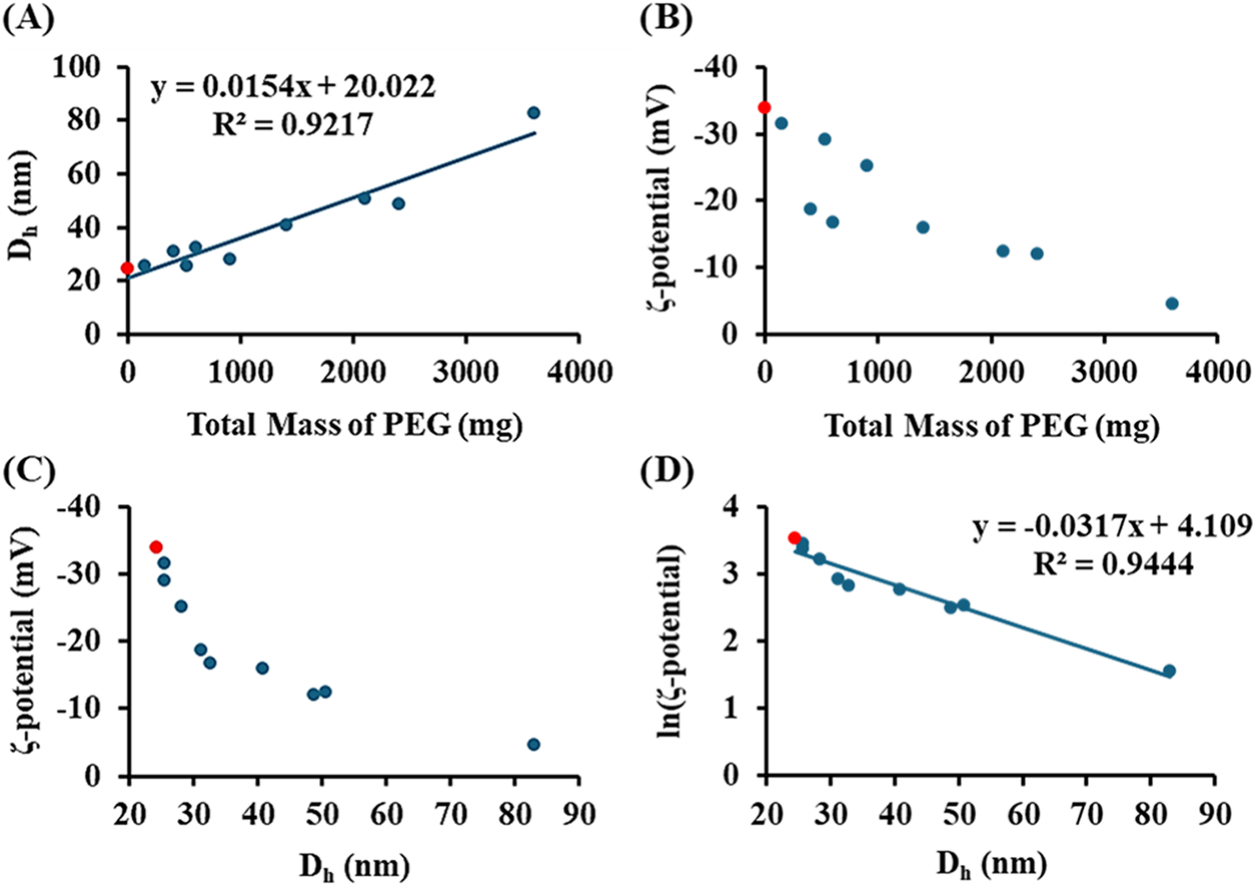
(A) Average *D*_h_ (*n* = 3) vs total mass of PEG, (B) average ζ-potential (*n* = 3) vs total mass of PEG, and (C) average ζ-potential vs average *D*_h_ (*n* = 3) across the GNPs-PEG series. (D) Linearized transform of average ζ-potential vs average *D*_h_ (*n* = 3) showing exponential fit.

Similar trends are documented in the literature for GNPs under various PEGylation methods, including physisorbed linear PEG, and extend to other metals/oxides, consistent with polymer-electrokinetic theory.^31,32,35–37^ Most pertinent to this experiment, Xue et al.^31^ reported a significant decrease in the magnitude of the ζ-potential range for GNPs with physisorbed OH-PEG–OH, Me-PEG–OH, and Me-PEG-Me preparations (−4.7 to −5.8 mV) when compared to the reported value for GNPs-citrate (−14 mV). It is commonly understood that PEG and other neutral polymer coatings (e.g., PVP) screen surface charge, thereby reducing the magnitude of ζ-potential relative to charge-stabilized (citrate) surfaces, while increasing *D*_h_.^38–40^ It has also been proposed for colloidal dispersions that increasing *D*_h_ via thicker/larger polymer layers shifts the slip plane outward, such that the ζ-potential at the plane is less negative; accordingly, the magnitude of ζ-potential decreases as *D*_h_ increases.^41,42^

The results of UV–vis characterization revealed a consistent λ_SPR_ across the GNPs-PEG series, indicating consistent GNPs-PEG core dimensions and suggesting that the substantial increase in *D*_h_ can be attributed to an increasing size of the physisorbed PEG coating.^43^ Slight red-shifting was observed for GNPs-PEG when compared to GNPs-citrate, mirroring well-documented observations for polymer-stabilized GNPs.^44–47^ The red shift is attributed directly to an increase in the refractive index near the GNP surface.^48,49^ The observed λ_SPR_ for GNPs-PEG was centered around ∼521 nm, consistent with what should be expected for GNPs in this size range.^50^ Representative UV–vis spectra for each molar ratio of the GNPs-PEG_6.0_ preparations are provided in Figure 3A–3C, accompanied by corresponding average λ_SPR_ values. The average experimental values for λ_SPR_ across the GNPs-PEG series are tabulated and plotted against *D*_h_ in the SI (Table S2, Figure S1). The results of ICP-MS characterization revealed, in all preparations of GNPs, a slight reduction in [Au] from the initial calculated concentration, as shown in Figure 3D (a table of the measured standard calibration curve data is provided in the SI, Table S3). These [Au] data from ICP-MS were used for calculating the [Au] of all GNPs-PEG preparations in the kinetic experiments and are reported in the SI (Table S4).

**Figure 3:**
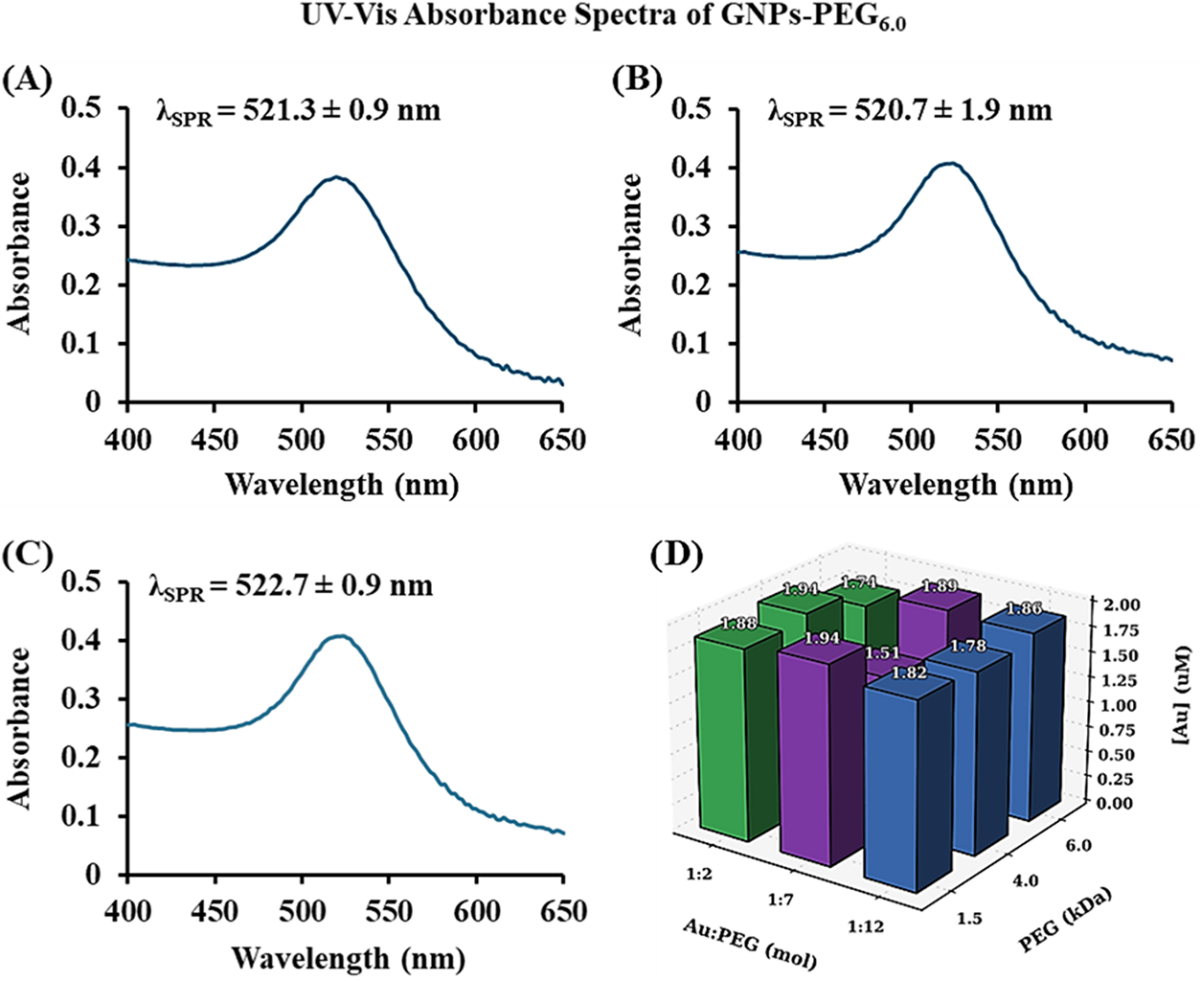
Representative UV–vis absorbance spectra showing average (*n* = 3) λ_SPR_ of (A) 1:2, (B) 1:7, and (C) 1:12 GNPs-PEG_6.0_ preparations. (D) ICP-MS determined [Au] concentrations across the GNPs-PEG series.

DLS and UV–vis were used to measure the *D*_h_, ζ-potential, and λ_SPR_ of GNPs-PEG*_n_* (1:12) samples after 6 months of storage at 4 °C. Representative plots for each of these measurements are provided in the SI (Figure S2). The results showed minimal changes in *D*_h_ values for all measured samples. All three replicates of each GNPs-PEG*_n_* (1:12) preparation showed similar peak *D*_h_ values as when they were originally measured, resulting in not only similar *D*_h_ values, but also standard deviations (SDs). The data also showed excellent ζ-potential preservation for these samples and no consistent directional trend for the observed shifts in value. GNPs-PEG_6.0_ (1:12) showed a right-shift, becoming slightly less negative, whereas GNPs-PEG_1.5_ (1:12) showed a left-shift, becoming slightly more negative. In contrast, GNPs-PEG_4.0_ (1:12) showed no shift at all. Similarly, the UV–vis data showed good preservation of λ_SPR_ and no consistent directional trend for the observed shifts.

Provided in the SI (Figure S3), additional characterization of GNPs-PEG_6.0_ (1:12) was collected via Fourier Transform Infrared Spectroscopy (FT-IR) and Transmission Electron Microscopy (TEM). The FT-IR analysis of the PEG-coated GNP sample showed characteristic PEG vibrational bands, including aliphatic C–H stretching near 2880 cm^–1^, CH_2_ bending/wagging modes in the 1460–1340 cm^–1^ region, and a strong ether C–O–C stretching envelope in the 1145–1060 cm^–1^ region. Together, these features support the adsorption of PEG on the GNPs.^51–53^ Furthermore, no strong carbonyl band was observed in the ∼1700 cm^–1^ region, indicating the absence of oxidized PEG. It should also be noted that the absence of an OH band is consistent with successful lyophilization and with the expected weak contribution of terminal hydroxyl groups in a long-chain polymer. As expected for a physisorbed PEG coating on citrate-capped GNPs, the FT-IR spectrum primarily reflects PEG-derived vibrational modes and serves as supporting evidence for successful PEG association. Figure S3A shows the FT-IR spectrum of GNPs-PEG_6.0_(1:12) overlaid on that of pure 6.0 kDa PEG (red line). Figure S3B shows a representative TEM image of this same GNPs-PEG_6.0_(1:12) sample with a 100 nm reference scale bar. As expected, the TEM instrument did not detect the polymer coatings,^54–56^ and the analysis showed metallic GNP cores with an average diameter of ∼20 nm, consistent with *D*_h_ evaluations of GNPs-citrate. Considering that the initial GNPs-citrate preparations were always synthesized with the same protocol, the consistency of the GNPs-citrate *D*_h_ measurements, and the TEM results, it is reasonable to approximate the thickness of PEG coatings throughout the series.

### Catalytic Activity of GNPs-PEG

Investigations into the catalytic activity of GNPs-PEG revealed a relatively consistent effective rate constant (*k*_eff_) across the GNPs-PEG*_n_* (*n* = 1.5, 4.0, and 6.0 kDa) series, when compared to chemically adsorbed counterparts.^57^ Figure 4 shows UV–vis kinetic profiles for the reduction of PNP catalyzed by three increasing Au concentrations ([Au] = 2.0, 4.0, and 8.0 μM) for each molar ratio of GNPs-PEG_6.0_ preparations. These time-dependent profiles show diminishing PNP peaks and increasing PAP peaks, as well as consistently faster reactions with increasing [Au]. In this way, 8.0 μM was determined to be the minimum [Au] at which complete reduction could be achieved within 60 s under these conditions, as seen in Figure 4. In addition, as shown in the SI (Figures S4–S6), a similar trend was observed for the *n* = 1.5 and 4.0 kDa PEG preparations and for GNPs-citrate.

**Figure 4:**
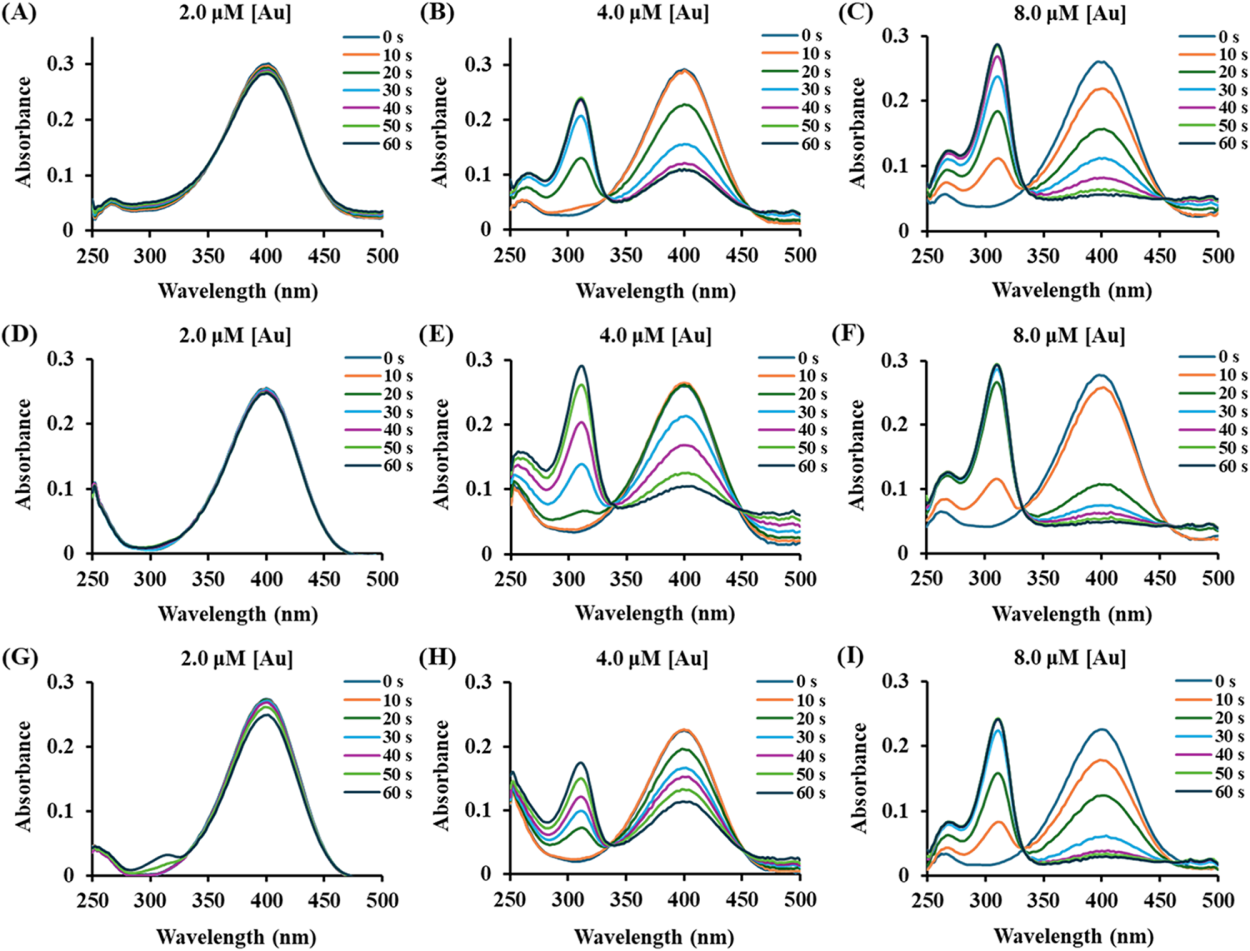
UV–vis kinetic profiles for the reduction of PNP to PAP by NaBH_4_ with increasing concentrations of GNPs-PEG_6.0_ preparations for (A–C) 1:2, (D–F) 1:7, and (G–I) 1:12 Au/PEG molar ratios.

The GNPs-PEG*_n_*-catalyzed PNP reduction reactions were performed in excess NaBH_4_, such that the *k*_eff_ values could be determined via pseudo-first-order kinetics. Figure 5 shows pseudo-first-order kinetic plots for each GNPs-PEG_6.0_ preparation, from which *k*_eff_ was determined by the slope (corresponding plots for *n* = 1.5, 4.0 kDa, and GNPs-citrate are provided in the SI, Figures S7–S9). The *k*_eff_ values associated with each preparation of GNPs-PEG*_n_*, across the series, are tabulated in Table 2 and are plotted against their corresponding total mass of PEG and *D*_h_ in Figure 6 (*k*_eff_ values for GNPs-citrate are indicated in red). The results show sustained catalytic activity across the GNPs-PEG*_n_* series.

**Figure 5:**
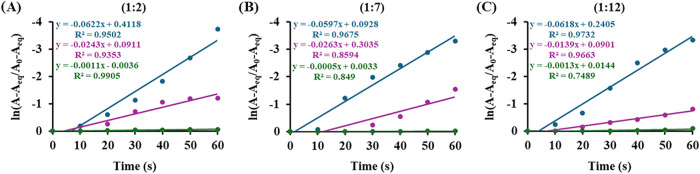
Pseudo-first-order kinetic plots of PNP reduction with 2.0 (green), 4.0 (purple), and 8.0 (blue) μM [Au] for (A) 1:2, (B) 1:7, and (C) 1:12 molar ratios of Au/PEG, for preparations with PEG_6.0_.

**Figure 6:**
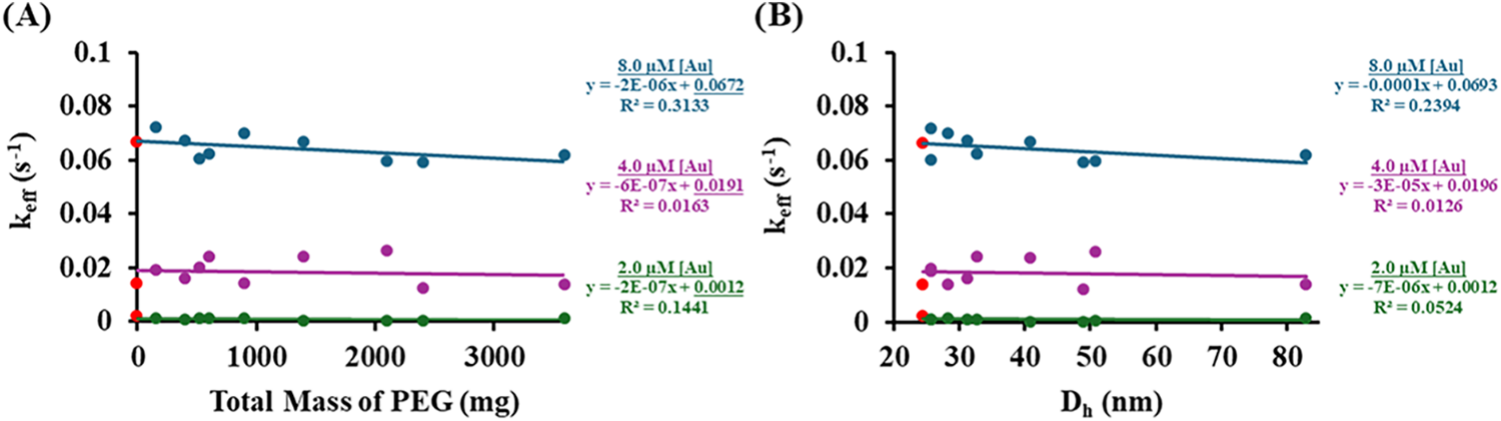
*k*_eff_ values vs (A) total mass of PEG and (B) *D*_h_, for 2.0 (green), 4.0 (purple), and 8.0 (blue) μM [Au], across the GNPs-PEG series (*k*_eff_ values for GNPs-citrate are indicated in red).

**Table 2:** Tabulated *k*_eff_ Values (± SE of Regression) at Each [Au] Concentration with CorrespondingGNPs-PEG*_n_* Preparations

GNPs-PEG*_n_* (Au/PEG)	2.0 μM [Au]	4.0 μM [Au]	8.0 μM [Au]
	*K*_eff_ × 10^–3^ s^–1^	*K*_eff_ × 10^–3^ s^–1^	K_*eff*_, × 10^–3^ s^–1^
GNPs-PEG_1.5_(1:2)	1.2 ± 0.2	19 ± 4	72 ± 4
GNPs-PEG_1.5_(1:7)	1.00 ± 0.06	20 ± 2	60 ± 4
GNPs-PEG_1.5_(1:12)	1.30 ± 0.05	14 ± 1	70 ± 7
GNPs-PEG_4.0_(1:2)	0.80 ± 0.08	16 ± 3	67 ± 2
GNPs-PEG_4.0_(1:7)	0.20 ± 0.05	24 ± 3	67 ± 8
GNPs-PEG_4.0_(1:12)	0.20 ± 0.04	12 ± 1	59 ± 2
GNPs-PEG_6.0_(1:2)	1.10 ± 0.05	24 ± 3	62 ± 6
GNPs-PEG_6.0_(1:7)	0.50 ± 0.09	26 ± 5	5.97 ± 5
GNPs-PEG_6.0_(1:12)	1.3 ± 0.3	14 ± 1	62 ± 0.46
GNPs-citrate	1.9 ± 0.4	14 ± 3	66 ± 7

The averages of *k*_eff_ across the GNPs-PEG series were 0.0008 ± 0.0004, 0.0189 ± 0.0048, and 0.0644 ± 0.0045 s^–1^ (*n* = 9, ± SD) for the 2.0, 4.0, and 8.0 μM [Au] series, respectively. Each average can be compared to its respective *k*_eff_ value for GNPs-citrate: 0.0019 ± 0.0004, 0.0139 ± 0.0032, and 0.0664 ± 0.0074 s^–1^ (±standard error of regression, SE). These data suggest that the catalytic activity of GNPs-citrate was effectively preserved across the GNPs-PEG series, with only a 3.1% difference between the GNPs-PEG average and GNPs-citrate, at 8.0 μM [Au]. It should be noted that this difference was 30.5% and 81.5% for 4.0 and 2.0 μM [Au], but these differences are associated with much more significant uncertainties. The relative standard deviations (%RSD) for the 2.0, 4.0, and 8.0 μM [Au] series were 49.7, 25.6, and 6.9%, indicating much higher reliability of the 8.0 μM [Au] data. In Figure 5 (and Figures S7–S9), the pseudo-first-order kinetic plots reveal lower *R* ^2^ values associated with the 4.0 and 2.0 μM [Au] kinetics data. The superior reliability of the 8.0 μM [Au] kinetics data was common across the GNPs-PEG series and can be attributed to several factors, the first of which is that it was the only [Au] at which PNP was completely reduced. The lower concentrations were far too slow, consequently providing fewer data points with which the reaction could be accurately modeled with first-order kinetics. Given the consistently superior reliability of the 8.0 μM [Au] kinetics data, these results were regarded with greater significance in the analysis and subsequently drawn conclusions.

The relationship between *k*_eff_ for GNPs-citrate and GNPs-PEG can be compared to many examples in the literature of GNPs under various PEGylation schemes. It is well documented that metallic NPs with chemically adsorbed polymer stabilizing systems, including but not limited to PEG, hyperbranched polymers (HBPs), and dendritic polymers, are subject to substantial loss of catalytic activity.^57–59^ This loss is most commonly attributed to a reduction in accessible catalytic surface area and the emergence of an induction period.^27,60^ Mechanistic explanations for the emergence of an induction period are still a topic of debate and exploration;^27^ however, it is commonly attributed to a ligand-induced diffusion barrier, which reduces the rate at which PNP can diffuse to the GNPs surface.^61–63^ Others have proposed that the induction period is a result of surface restructuring by adsorbed reactant molecules.^23,64^ In 2020, Neal et al. attributed the induction period largely to dissolved O_2_ in air-equilibrated reaction media and showed that chemically bound stabilizing ligands can prolong this period by slowing O_2_ consumption before PNP reduction begins.^28^ Regardless of the precise mechanistic explanation for the induction period, its corresponding emergence with NPs stabilized by chemisorbed polymers is documented repeatedly in the literature.

Beyond the induction period, chemisorbed polymers have been shown to continue the suppression of catalytic activity, thus reducing *k*_eff_. Ansar and Kitchens^27^ demonstrated an inverse relationship between the *k*_eff_ of PNP reduction and the grafting density of thiol-terminated PEG (PEG*_n_*-SH) to the GNPs surface. They showed a clear reduction in *k*_eff_ (2.1, 0.28, 0.15, and 0.06 min^–1^) for GNPs with chemically adsorbed PEG_30.0, 10.0, 5.0, 2.0_-SH, corresponding to a decrease in the available catalytic surface area relative to GNPs-citrate (84, 54, 27, and 9%). They also showed that all these *k*_eff_ values were lower than their reported value for GNPs-citrate (2.3 min^–1^). Que et al.^57^ reported similar results for GNPs with chemically adsorbed PEG-*b*-polystyrene (PEG-*b*-PS). Across a series of preparations with increasing polystyrene chain length, they reported GNPs-citrate to have the highest *k*_eff_ (4.6 × 10^–3^ s^–1^), compared to lower and decreasing values (2.2 × 10^–3^, 1.9 × 10^–3^, and 1.5 × 10^–3^ s^–1^) for PEG-*b*-PS. They also explicitly state that the catalytic activity of GNPs with PEG-SH coatings was the lowest of all. For these, they report only 60% reduction of PNP after 1 h, but do not provide a specific *k*_eff_. Similarly, Chakraborty and Kitchens^65^ reported an inverse relationship between the *k*_eff_ of PNP reduction and the grafting density of thiol-terminated poly­(acrylic acid) (SPAA) to the GNPs surface. They explicitly state that their measured *k*_eff_ value for GNPs-citrate (0.66 min^–1^) was higher than any of the SPAA functionalized preparations, “depicting expected surface passivation.” Their reported pseudo-first-order kinetic plots show substantial decreases in *k*_eff_, corresponding to higher SPAA contents, though they do not provide specific *k*_eff_ values for the SPAA functionalized GNPs.

In contrast to these chemically adsorbed PEG coatings, the consistency of *k*_eff_ across the GNPs-PEG series and its similarity to that of GNPs-citrate strongly support the conclusion that physisorbed, linear OH-PEG–OH does not impose a significant consequence to catalytic activity. The aforementioned 3.1% difference between the *k*_eff_ for GNPs-citrate and the average *k*_eff_ for GNPs-PEG (at 8.0 μM [Au]) can be compared to the corresponding differences of 112.1% and 84.5% for the data reported by Ansar and Kitchens^27^ and Que et al.,^57^ respectively. Furthermore, the differences between their reported values for GNPs-citrate and those of their most catalytically suppressed preparations of GNPs-PEG*_n_* (-SH and -PS) were 189.8% and 101.6%, respectively. These can be compared to the largest individual difference of only 11.3%, reported here in the 8.0 μM [Au] data (GNPs-PEG_4.0_ (1:12)). This difference is even smaller (7.2%) for GNPs-PEG_6.0_ (1:12), the preparation with the largest associated *D*_h_ and highest total mass of PEG.

To further justify this conclusion, one-sample *t* tests with 95% confidence intervals (CI) and control charts were performed, analyzing *k*_eff_ ratios (GNPs-PEG*_n_*/GNPs-citrate) at each [Au]. The *t* tests were performed using mean log ratios (*r̅*), according to eqs 1–3, ensuring symmetry around 1.0 and satisfying normality assumptions of the *t* test. The resulting CI bounds were then back-transformed to the original ratio-scale for reporting (*e* ^lower^, *e* ^upper^).

1 $$r_{i}=\ln\left(\frac{k_{{\mathrm{eff}}_{i}}}{k_{\mathrm{eff},\mathrm{cit}}}\right)$$

2 $$\mathrm{r̅}=\frac{1}{n}\sum_{i=1}^{n}r_{i}$$

3 $$\mathrm{CI}=\mathrm{r̅}\pm t_{\alpha /2,\mathrm{df}=n-1}\cdot \frac{\mathrm{SD}}{\sqrt{n}}$$

In accordance with this *t* test structure, the null hypothesis was defined as H_0_: *r̅* = 1 (log-scale, *r̅* = 0). Consequently, the alternative hypothesis was defined, H_a_: *r̅* ≠ 1 (log-scale, *r̅* ≠ 0). The calculated CI for the 8.0 μM [Au] series was 0.915–1.023, representing the range of parameter values that would fail to reject H_0_ in a standard 2-tailed *p*-value *t* test (*p* > 0.05). If the null value lies within this range, the data fail to reject H_0_, indicating no significant difference between the average *k*_eff_ of GNPs-PEG*_n_* and the *k*_eff_ of GNPs-citrate. Unsurprisingly, at a 95% CI (*t*_0.975, 8_ = 2.306), only the *k*_eff_ values for the 8.0 μM [Au] series failed to reject the null hypothesis. Furthermore, this CI lies well within a 10% practical equivalence margin (0.905–1.105; log-symmetric). The CIs for the 2.0 and 4.0 μM [Au] series were 0.202–0.653 and 1.062–1.626, respectively, both clearly rejecting the null hypothesis and indicating a significant difference. Figure 7 shows control charts for each [Au]. These plot *k*_eff_ ratios of GNPs-PEG*_n_*/GNPs-citrate against the indexed GNPs-PEG*_n_* preparations and visually illustrate the consistency of the data around constant markers: perfect equivalence, mean, and ± 2 standard deviations (σ).

**Figure 7:**
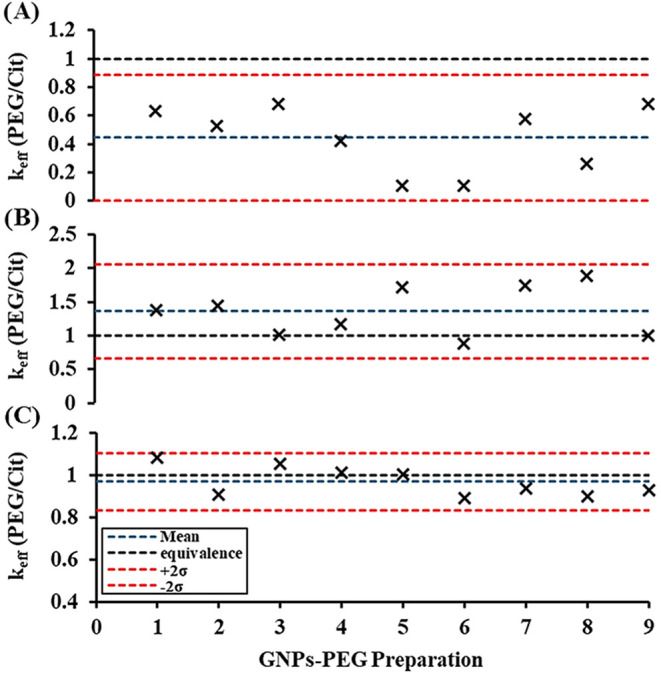
Control charts of *k*_*eff*_ ratios at (A) 2.0, (B) 4.0, and (C) 8.0 μM [Au], with mean, equivalence, and ± 2σ constants.

### Stability of GNPs-PEG*_n_* (Au/PEG)

The stability of GNPs-PEG in the PNP reduction medium was investigated by measuring catalytic activity retention over the course of nine sequential substrate additions. After an initial reaction, identical to those discussed in the catalytic activity section, nine consecutive reactions were performed via the addition of 20 μL spikes of 1.7 mM PNP to that same reaction medium (with already present GNPs-PEG). The successive reactions were performed only after all the PNP of the previous reaction was reduced, indicated by the flattening of the PNP absorbance peak. Figure 8 shows plots of the normalized absorbance vs time data for each subsequent reaction and the corresponding pseudo-first-order kinetic plots, from which the *k*_eff_ values provided in Table 3 were determined. Figure 9 shows the percent catalytic activity of GNPs-PEG_6.0_ (1:12) over the course of these 10 additions, where the *k*_eff_ of the initial reaction is 100%

**Figure 8:**
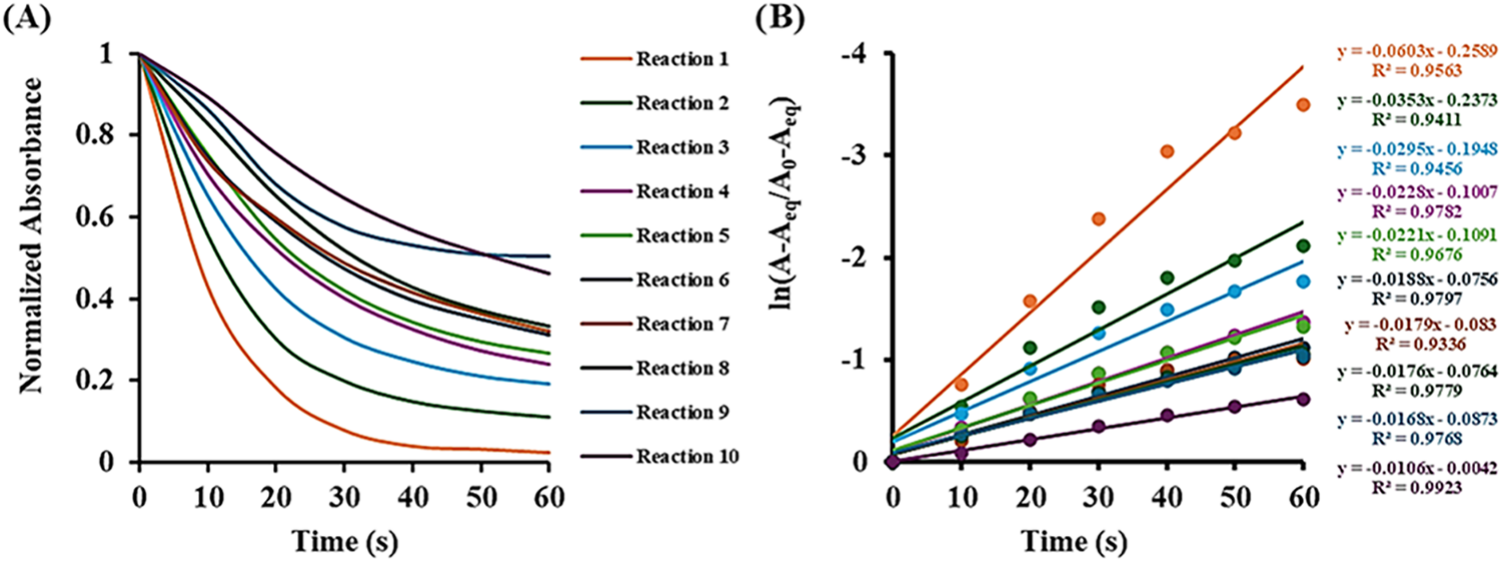
Normalized and baseline-corrected absorbance vs time plots (A) and corresponding pseudo-first-order kinetic plots (B) for 10 consecutive PNP reductions with GNPs-PEG_6.0_ (1:12).

**Figure 9:**
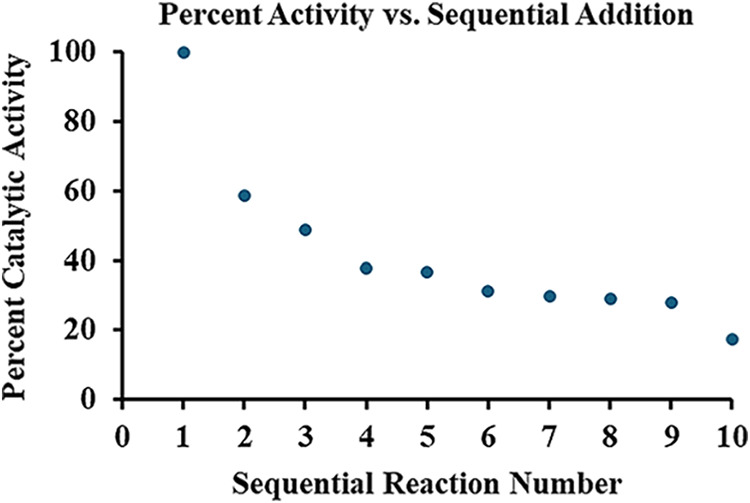
Percent catalytic activity of GNPs-PEG_6.0_ (1:12) over 10 consecutive reactions.

**Table 3:** Tabulated *k*_eff_ Values (±SE) for 10 Consecutive PNP Reductions with GNPs-PEG_6.0_ (1:12)

reaction number	*k*_eff_, × 10^–3^ s^–1^
1	60 ± 6
2	35 ± 4
3	30 ± 3
4	23 ± 2
5	22 ± 2
6	19 ± 1
7	18 ± 2
8	18 ± 1
9	17 ± 1
10	11.0 ± 0.4

The data showed, with good reliability (strong *R* ^2^ values and small standard errors), a consistent decline in *k*_eff_ over the course of reactions. These data agree with previous reports and with what should be expected when considering the results of the catalytic activity section. This loss of catalytic activity, for GNPs with various stabilizing systems (including chemically adsorbed PEG-SH), is documented in the literature^66^ and commonly attributed to the partial aggregation of GNPs in NaBH_4_.^65–67^ Using this sequential PNP addition method, Harrison et al.^66^ show a similar plot shape to that of Figure 9. They show a sharp decline in catalytic activity from the first to the second reaction, followed by a more steadily declining activity for the rest of the reactions. They also report explicitly that PEG-SH functionalized GNPs showed the best retention of catalytic activity, at the cost, however, of “initial apparent kinetics.”

The stability of GNPs-PEG was further investigated by measuring the *D*_h_ of the same GNPs-PEG_6.0_ (1:12) sample in the reduction medium over time, via DLS. Consistent with expected PEG desorption, the results (SI, Figure S10) showed increasing peak-*D*_h_ values and increasing *D*_h_ distributions, indicating GNP aggregation over the course of 25 min. This 25 min window was comparable to the time required to run the 10 sequential reactions discussed above (30 min). Additionally, a stark color change was observed from the characteristic ruby-red color of stable GNPs to a dark black/gray color, further signaling that the stabilizing PEG coatings had desorbed, and significant aggregation had occurred.

Collectively, the results presented herein indicate that increasingly large, physisorbed PEG coatings were successfully formed around GNPs-citrate and that they do not induce a statistically significant reduction in initial catalytic activity. This preservation of activity includes the absence of an observed induction period. Additionally, they were shown to effectively preserve their dimensional properties and colloidal stability over time. Furthermore, as indicated by the results of the stability assays in the PNP reduction medium and consistent with many reports in the literature,^28–30,68,69^ the physisorbed PEG coating likely desorbs much more efficiently in NaBH_4_ than chemically adsorbed counterparts, thus inducing no significant consequence to the initial catalytic activity of GNPs-citrate. Ansar et al.^29^ and Neal et al.^28^ reported that even strongly bound organothiols show complete desorption in aqueous NaBH_4_. They assert that hydride ions (H^–^) effectively displace organic ligands on the Au surface. However, the reduced initial activity of these systems suggests that organothiol desorption is slow enough for surface passivation and diffusion barriers to persist, producing induction periods and attenuated performance. In fact, Neal et al. showed that ligand desorption times scale with bond dissociation energy, the longest reported time being 10 min for a thiolated phenol. These results ultimately constitute an early stage concept validation and demonstrate the feasibility of an ISSS.

## Conclusions

This work demonstrates a strategy to decouple long-term colloidal stability from initial catalytic performance in aqueous GNPs by using a labile, physisorbed polymer coating. Across the investigated PEG molecular weights and loadings, physisorbed hydroxyl-terminated PEG produced clear evidence of polymer association with GNPs-citrate, including increased *D*_h_ and reduced magnitude of ζ-potential, while leaving the plasmonic response largely unchanged. These observations are consistent with preservation of the metallic core and formation of a neutral interfacial layer. Most importantly, these coatings did not measurably suppress the initial catalytic performance of GNPs-citrate in the NaBH_4_ reduction of PNP, and no induction period was observed. This contrasts with the reactivity commonly associated with strongly chemisorbed polymeric stabilizers.

Beyond initial performance, the coating provided meaningful benefit during storage. GNPs-PEG maintained key colloidal and optical metrics over an extended period of storage, supporting the premise that physisorbed PEG can function as a storage-stabilizing layer. Under reducing reaction conditions, time-dependent loss of stability and activity was observed alongside increasing *D*_h_, consistent with destabilization driven by the removal or disruption of the physisorbed layer in NaBH_4_-containing media. In combination with prior literature describing ligand loss under borohydride conditions, these observations support the central ISSS concept of a stabilizing layer that is effective during storage and handling yet sufficiently labile under catalytic conditions to preserve initial activity.

Future work should interrogate postreaction morphology and interfacial chemistry using TEM and complementary spectroscopies. In addition, kinetic evaluations of an ISSS should be extended to higher-stress environments relevant to practical catalysis, including elevated ionic strength, broader pH windows, mixed-solvent systems, higher temperatures, and alternative reductants or reactant matrices, to map where physisorbed stabilization remains beneficial versus where it fails and why. Furthermore, it would be valuable to study the colloidal stability of these physisorbed GNPs-PEG systems in higher-stress dispersion media, such as salt solutions and bacterial media.

## Experimental Section

### Materials

Linear hydroxyl-terminated poly­(ethylene) glycol (OH-PEG–OH; 1.5, 4.0, and 6.0 kDa), tetrachloroauric­(III) acid trihydrate (HAuCl_4_·3H_2_O, ≥ 49% Au), sodium citrate dihydrate (Na_3_C_6_H_5_O_7_·2H_2_O), sodium hydroxide (NaOH, ≥ 97%), sodium borohydride (NaBH_4_, 99%), and trace metal grade nitric acid (HNO_3_, 67–70%, w/w, 30–33%) were obtained from Fisher Scientific. *p*-Nitrophenol (PNP) was obtained from Acros Organics (99%). Au ICP standard (100 mg L^–1^, 2% HCl) was obtained from SPEX CertiPrep.

### Instrumentation

Hydrodynamic diameter (*D*_h_, nm) and zeta potential (ζ, mV) were measured on a Malvern Zetasizer ZS90 dynamic light scattering (DLS) instrument (He–Ne, 90°; Malvern Panalytical, U.K.) using the manufacturer’s Zetasizer software. UV–visible (UV–vis) spectra and kinetic traces were collected on a Thermo Fisher GENESYS 150 spectrophotometer (1.0 cm disposable cuvettes; Thermo Fisher Scientific). Gold concentrations ([Au]) were determined by Agilent 7900 ICP-MS (indium internal standard, argon plasma, HNO_3_ wash; Agilent Technologies) with data acquisition and quantitation in MassHunter Workstation v4.4. Fourier transform infrared (FT-IR) spectra were collected on a Bruker α-P attenuated total reflectance (ATR) FT-IR spectrometer (Bruker Corporation) with data acquisition and quantitation in Opus v6.5. Transmission electron microscopy (TEM) images were collected with a JEOL-JEM 2100 electron microscope.

### GNPs-Citrate Synthesis

Citrate-stabilized GNPs (GNPs-citrate) were synthesized following the Turkevich method.^7^ In brief, 5.0 mL of 10.0 mM HAuCl_4_·3H_2_O was added to 45.0 mL of deionized (DI) water ([Au] = 1.0 mM). The solution was then heated to 95–100 °C and stirred at 1100 rpm on a hot plate. After achieving a gentle and uniform boil, 29.4 mg of Na_3_C_6_H_5_O_7_·2H_2_O, dissolved in a 3.0 mL aliquot of DI water (33.3 mM), was rapidly injected into the stirring solution (1:2 molar ratio, Au/Na_3_C_6_H_5_O_7_). After observing the characteristic color change of yellow to deep “ruby red,” the solution was allowed to continue stirring at a gentle boil for 20 min. The solution was then transferred from the hot plate to a stir plate (700 rpm) and allowed to cool to room temperature.

### Preparation of GNPs-PEG

A series of GNPs with physisorbed OH-PEG–OH (PEG: 1.5, 4.0, 6.0 kDa) coatings was prepared (GNPs-PEG*_n_*). A variable mass of PEG was added to the previously synthesized GNPs-citrate solution and allowed to stir for 1 h at room temperature (1100 rpm). Each molecular weight of PEG was used to prepare samples containing 1:2, 1:7, and 1:12 molar ratios of Au to PEG. The product was then collected as-is in a foil-wrapped centrifuge tube for subsequent testing and storage. Furthermore, triplicate samples for each ratio, at each molecular weight of PEG, were prepared and tested independently. All characterization values are reported as mean ± SD (*n* = 3). The total mass of PEG used in each preparation is provided in Table 1.

### DLS Characterization

Hydrodynamic diameter (*D*_h_) was measured under the following parameters: 1.0 cm disposable cuvette, GNPs analyte (0.468 absorption, 0.647 refractive index), water dispersant (0.8872 cP viscosity, 1.330 refractive index), 15 runs, 10-s run time, 25 °C, 120-s equilibration time. Zeta potential (ζ) was measured under the following parameters: DTS1070 Malvern Folded Zeta Capillary Cell, GNPs analyte (0.468 absorption, 0.647 refractive index), water dispersant (0.8872 cP viscosity, 1.330 refractive index, 78.5 dielectric constant), 15 runs, 25 °C, 120-s equilibration time.

### UV–vis Characterization

UV–vis absorbance spectra were collected for each sample to measure the surface plasmon resonance (λ_SPR_) of GNPs (400–800 nm range, 2.0 nm step). Samples were diluted in 3.0 mL of DI water to 11.0% by volume.

### ICP-MS Characterization

The [Au] of each GNPs-PEG*_n_* (Au/PEG) sample was determined by ICP-MS. Samples were diluted to 2.5 μM (0.5 ppm) in DI water, assuming an initial [Au] of 1 mM (∼197 ppm), and prepared in 2.0% trace metal grade HNO_3_. Each sample was measured with a 6-point standard curve composed of a blank and 0.05, 0.25, 0.51, 2.5, and 5.1 μM [Au] standards (0.01, 0.05, 0.1, 0.5, and 1.0 ppm). Each standard was also prepared in 2.0% trace metal grade HNO_3_. All the samples prepared in this experiment were placed in an autosampler rack and measured in the same run, using a predefined sampling method. The method included 3 blank measurements, 1 measurement of each [Au] standard, and 1 HNO_3_ wash, prior to any measurements of the GNPs-PEG*_n_* (Au/PEG) samples. Additionally, the samples were measured in groups of 6, between which there were two quality control measurements using the [Au] standards and an HNO_3_ wash.

### FT-IR Characterization

FT-IR spectra were collected for both GNPs-PEG_6.0_(1:12) and pure 6.0 kDa hydroxylated PEG. Prior to FT-IR data acquisition, the GNPs-PEG_6.0_(1:12) sample was stored in a −20 °C freezer for 24 h. The sample was then lyophilized for 48 h, producing a freeze-dried sample, free of water. The pure hydroxylated PEG was unaltered and analyzed as-is from the manufacturer.

### TEM Imaging

GNPs were adhered to a freshly glow-discharged and carbon-coated 200-mesh copper EM grid (Electron Microscopy Sciences, EMS) by adding 5 μL of GNPs suspended in KHE buffer. A drop of 2% aqueous uranyl acetate negative stain solution (EMS) was then added to the EM grid, and any excess stain solution was gently wicked away using Whatman filter paper. The TEM images were captured using a JEOL-JEM 2100 electron microscope.

### Reaction Kinetics: Reduction of PNP

The catalytic activities of GNPs-citrate and GNPs-PEG*_n_* (Au/PEG) were investigated by collecting UV–vis spectra (250–500 nm range, 2.0 nm step) at 10-s intervals over the course of a PNP reduction reaction. Cuvettes were prepared with 170.0 μL of 0.2 mM PNP, 1.0 mL of 150.0 mM NaBH_4_, and 1.53 mL of pH 11 water (NaOH in DI). Informed by the results of ICP-MS, GNPs were tested in this environment at three increasing [Au]: 2.0, 4.0, and 8.0 μM. Each kinetic trace was blanked, prior to the reaction, with a separate cuvette containing 2.7 mL of pH 11 water and a variable concentration of GNPs, corresponding to the subsequent reduction. Effective rate constants (*k*_eff_, s^–1^) were calculated from these data using the PNP peak and eq 4,^70^ where *A*_*t*_ is the absorbance at time *t*, *A*_0_ is the initial absorbance, and *A*_*∞*_ is the absorbance at equilibrium.

4 $$\ln\frac{A_{t}-A_{\infty }}{A_{0}-A_{\infty }}=-k_{\mathrm{eff}}t$$

*A* values were determined as the maximum absorbance of the PNP peak at a given *t*, *A*_0_ values were determined as the maximum absorbance of the PNP peak at *t* = 0, and *A*_*∞*_ values were determined as the average absorbance of the plateau region for each interval of *t* (λ = 480–500 nm). All absorbance data were normalized to *A*_0_, such that *A*_0_ = 1. The uncertainty of *k*_eff_ is reported as ± the standard error of regression analysis (SE).

### Stability Tests of GNPs-PEG

The stability of GNPs-PEG in the PNP reduction medium was investigated using the same reaction conditions and *k*_eff_ analysis as previously described. After an initial reaction, sequential 20.0 μL spikes of 1.7 mM PNP were added to the cuvette, and the consecutive reactions were measured with already present GNPs-PEG_6.0_ (1:12). Successive spikes of PNP were only added after *A* was equal to *A*_*∞*_, indicating that all the PNP had been reduced.

The stability of GNPs-PEG in the PNP reduction medium was further investigated by measuring *D*_h_ over time, using DLS. To the same reaction conditions previously described, 20 μL of GNPs-PEG_6.0_ (1:12) was added, and the *D*_h_ was recorded at 4 intervals of time over the course of 25 min. The instrumental parameters of the DLS were the same as previously described.

## Supplementary Material



## Notes

Includes DLS and SPR characterization data for characterization and stability; TEM and FT-IR for further characterization; and detailed kinetics data for the reduction reaction (PDF)
