# Supplementary material for: Decoupling Colloidal Stability and Catalytic Activity of Gold Nanocatalysts via In-Situ Sacrificial Physisorbed PEG Coatings
Source: Langmuir. 2026 Jun 2;42(23):17060–71. doi: 10.1021/acs.langmuir.6c01965 (PMC13276891; doi:10.1021/acs.langmuir.6c01965)
Supplement: Supplementary file 1 [file la6c01965_si_001.pdf]

“Supporting Information”

Decoupling Colloidal Stability and Catalytic Activity of Gold Nanocatalysts via In-Situ Sacrificial Physisorbed PEG Coatings

Authors: Andrew A. Pettenger, Shayd Gothard, Tuhina Banerjee, Santimukul Santra*

Department of Chemistry and Biochemistry, Missouri State University, 901 S. National Avenue, Springfield, MO 65897, USA

*Corresponding author: Santimukul Santra. Email: ssantra@missouristate.edu

TABLE OF CONTENTS **Page No.**

Table S1: Characterization: tabulated experimental values of D_h and ζ-potential……………..… **S2**
Table S2: Characterization: tabulated experimental values of λ_{SPR}……………………………... **S3**
Table S3: Characterization: tabulated ICP-MS standard calibration curve data………………... **S3**
Table S4: Characterization: tabulated ICP-MS-measured [Au] data…………………………… **S4**
Figure S1: Characterization: experimental values of λ_{SPR} plotted against corresponding D_h…….**S4**
Figure S2: Characterization: D_h, ζ-potential, and λ_{SPR} plots after 6 months in storage………… **S5**
Figure S3: Kinetics Data: UV-Vis kinetic profiles of PNP reduction at increasing [Au] for GNPs-PEG$_{1.5}$………………………………………………………………………………… **S6**
Figure S4: Kinetics Data: UV-Vis kinetic profiles of PNP reduction at increasing [Au] for GNPs-PEG$_{4.0}$………………………………………………………………………………… **S7**
Figure S5: Kinetics Data: UV-Vis kinetic profiles of PNP reduction at increasing [Au] for GNPs-citrate………………………………………………………………………………… **S7**
Figure S6: Kinetics Data: Pseudo first-order kinetic plots at increasing [Au] for GNPs-PEG$_{1.5}$ **S8**
Figure S7: Kinetics Data: Pseudo first-order kinetic plots at increasing [Au] for GNPs-PEG$_{4.0}$ **S8**
Figure S8: Kinetics Data: Pseudo first-order kinetic plots at increasing [Au] for GNPs-citrate **S8**
Figure S9: Stability Data: D_h measurements over time in PNP reduction medium…………… **S9**

CHARACTERIZATION

Table S1. Tabulated averages (n = 3; ± SD) of D_h and ζ-potential characterization data, with corresponding GNPs preparations.

GNPs-PEG$_n$(Au:PEG)	D_h (nm)	ζ-potential (mV)
GNPs-PEG$_{1.5}$(1:2)	26 ± 2	-32 ± 1
GNPs-PEG$_{1.5}$(1:7)	26 ± 2	-29 ± 1
GNPs-PEG$_{1.5}$(1:12)	28 ± 1	-25 ± 2
GNPs-PEG$_{4.0}$(1:2)	31 ± 2	-19 ± 1
GNPs-PEG$_{4.0}$(1:7)	41 ± 3	-16 ± 1
GNPs-PEG$_{4.0}$(1:12)	49 ± 7	-12 ± 1
GNPs-PEG$_{6.0}$(1:2)	33 ± 1	-17 ± 2
GNPs-PEG$_{6.0}$(1:7)	51 ± 1	-13 ± 2
GNPs-PEG$_{6.0}$(1:12)	83 ± 6	-5 ± 1
GNPs-citrate	24 ± 1	-34 ± 2

Table S2. Tabulated averages (n = 3; ± SD) of λ_{SPR} characterization data, with corresponding GNPs preparations.

GNPs-PEG$_n$(Au:PEG)	λ_{SPR} (nm)
GNPs-PEG$_{1.5}$(1:2)	521 ± 1
GNPs-PEG$_{1.5}$(1:7)	523 ± 1
GNPs-PEG$_{1.5}$(1:12)	520 ± 2
GNPs-PEG$_{4.0}$(1:2)	523 ± 1
GNPs-PEG$_{4.0}$(1:7)	524 ± 2
GNPs-PEG$_{4.0}$(1:12)	525 ± 3
GNPs-PEG$_{6.0}$(1:2)	521 ± 1
GNPs-PEG$_{6.0}$(1:7)	521 ± 2
GNPs-PEG$_{6.0}$(1:12)	523 ± 1
GNPs-citrate	520 ± 2

Table S3. Tabulated ICP-MS standard calibration curve data.

Standard	[Au] Theoretical (PPM)	[Au] Theoretical (µM)	[Au] Calculated (µM)	Counts Per Second (CPS)
1 (blank)	0	0	0	-13
2	0.01	0.05	0.01	52,656
3	0.05	0.25	0.10	426,476
4	0.1	0.51	0.24	1,009,014
5	0.5	2.5	2.47	10,251,277
6	1	5.1	5.14	20,821,633

Table S4. Tabulated averages (n = 3; ± SD) of [Au] characterization data for original dilutions based on ICP-MS experiments.

**GNPs-PEG$_n$(Au:PEG)**	**[Au] (mM)**
GNPs-PEG$_{1.5}$(1:2)	0.74 ± 0.18
GNPs-PEG$_{1.5}$(1:7)	0.76 ± 0.01
GNPs-PEG$_{1.5}$(1:12)	0.72 ± 0.03
GNPs-PEG$_{4.0}$(1:2)	0.76 ± 0.02
GNPs-PEG$_{4.0}$(1:7)	0.60 ± 0.16
GNPs-PEG$_{4.0}$(1:12)	0.70 ± 0.06
GNPs-PEG$_{6.0}$(1:2)	0.68 ± 0.07
GNPs-PEG$_{6.0}$(1:7)	0.74 ± 0.03
GNPs-PEG$_{6.0}$(1:12)	0.73 ± 0.03
GNPs-citrate	0.65 ± 0.17

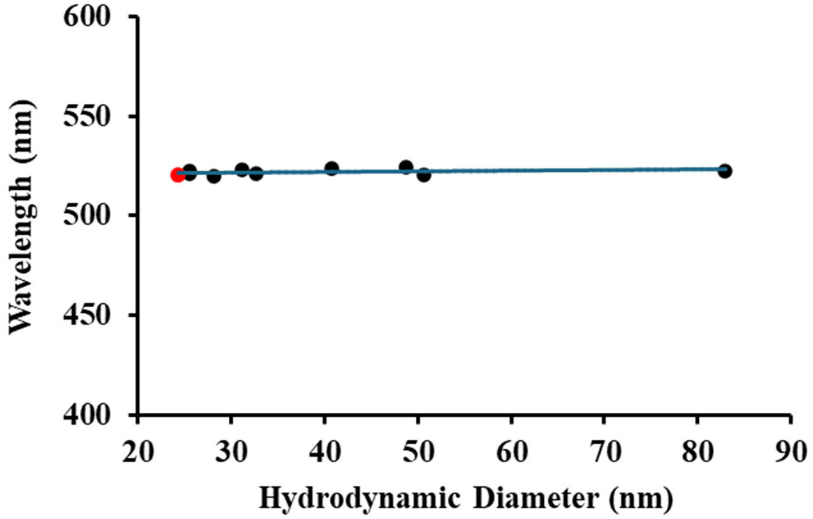

Figure S1. Average λ_{SPR} (n = 3) plotted against average D_h (n = 3) across the GNPs-PEG series. λ_{SPR} of GNPs-citrate is indicated in red.

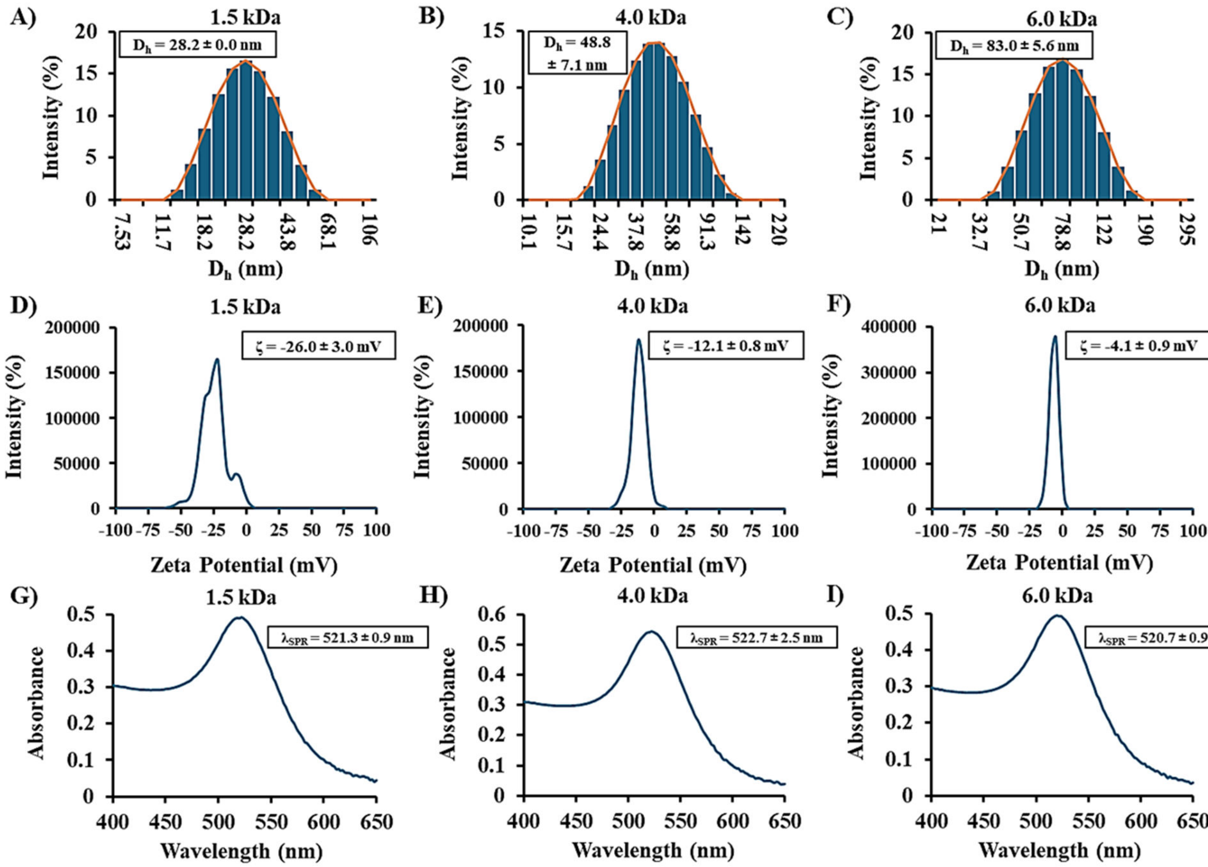

Figure S2. Representative plots for (**A**) D$_h$, (**B**) ζ-potential, and (**C**) λ_{SPR} measurements of GNPs-PEG$_n$ (1:12) samples, after 6 months of storage. Corresponding values reported as mean ± SD (n = 3).

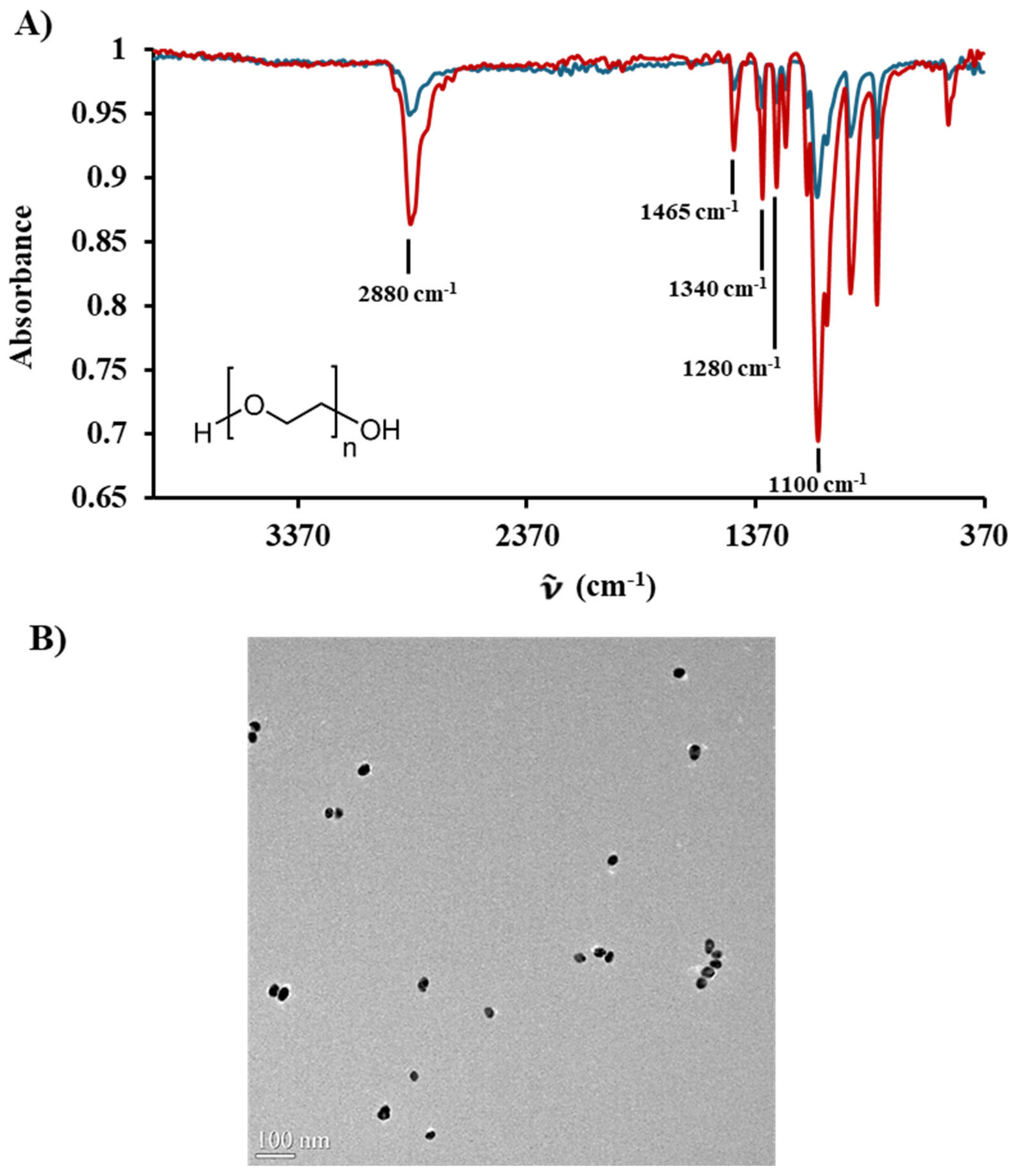

Figure S3. (A) FT-IR spectrum of GNPs-PEG$_{6.0}$(1:12) overlayed on that of pure 6.0 kDa hydroxylated PEG with labeled peaks. (B) TEM image of GNPs-PEG$_{6.0}$(1:12) with 100 nm reference scale.

KINETICS DATA

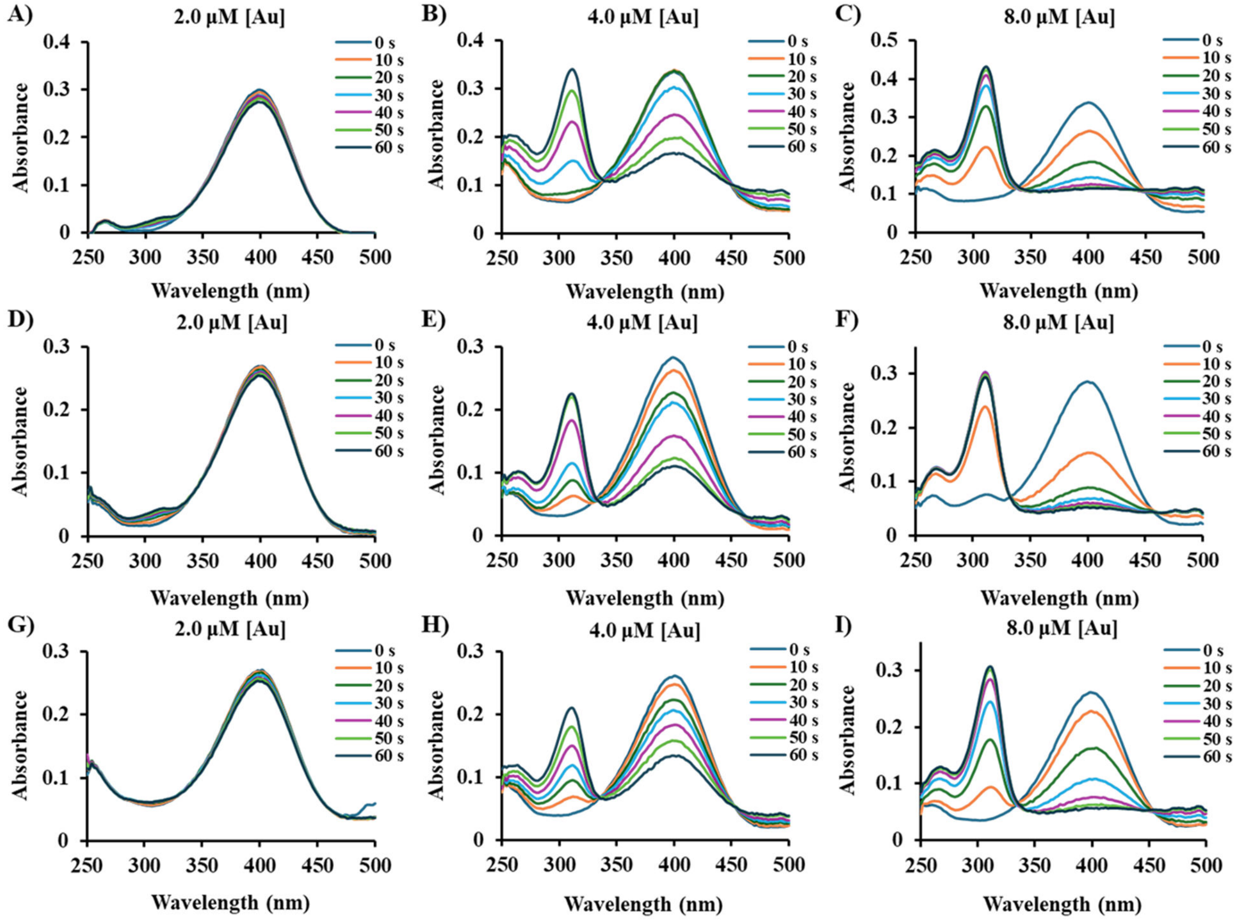

Figure S4. UV-Vis kinetic profiles for the reduction of PNP to PAP by $NaBH_4$, with increasing concentrations of GNPs-$PEG_{1.5}$ preparations at (**A-C**) 1:2, (**D-F**) 1:7, and (**G-I**) 1:12 Au:PEG molar ratios.

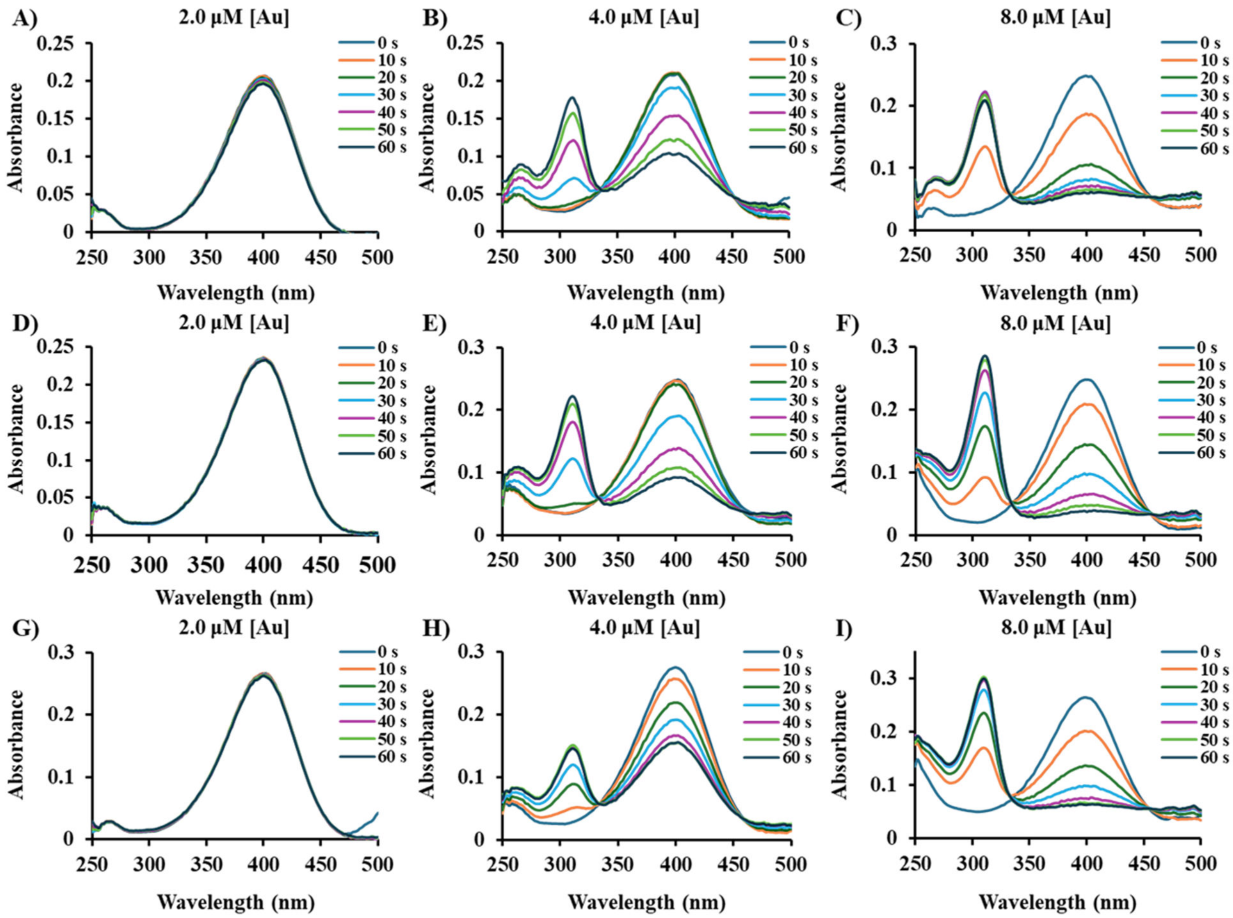

Figure S5. UV-Vis kinetic profiles for the reduction of PNP to PAP by $NaBH_4$, with increasing concentrations of GNPs-$PEG_{4.0}$ preparations at (**A-C**) 1:2, (**D-F**) 1:7, and (**G-I**) 1:12 Au:PEG molar ratios.

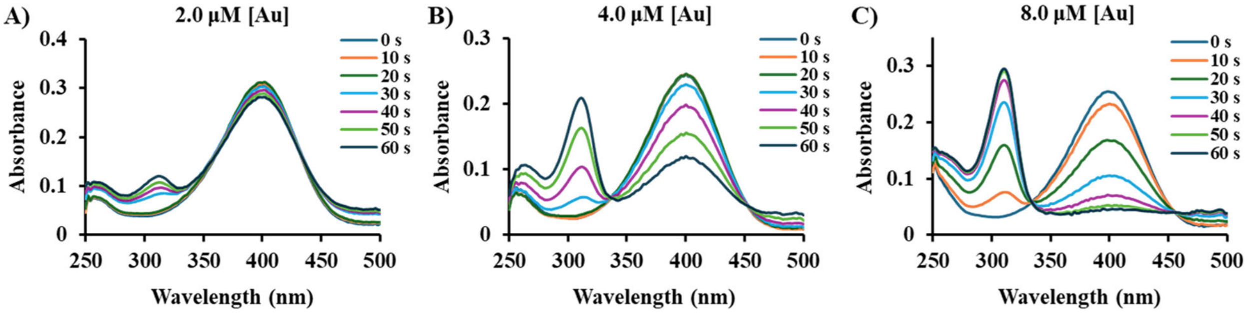

Figure S6. UV-Vis kinetic profiles for the reduction of PNP to PAP by $NaBH_4$, with increasing concentrations of GNPs-citrate.

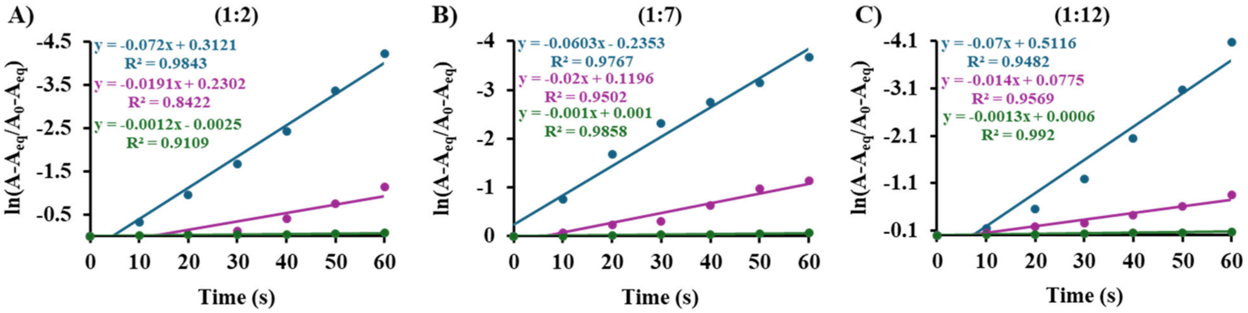

Figure S7. Pseudo first-order kinetic plots of PNP reduction with 2.0 (green), 4.0 (purple), and 8.0 (blue) μM [Au] for (A) 1:2, (B) 1:7, and (C) 1:12 molar ratios of Au:PEG, for preparations with GNPs-$PEG_{1.5}$.

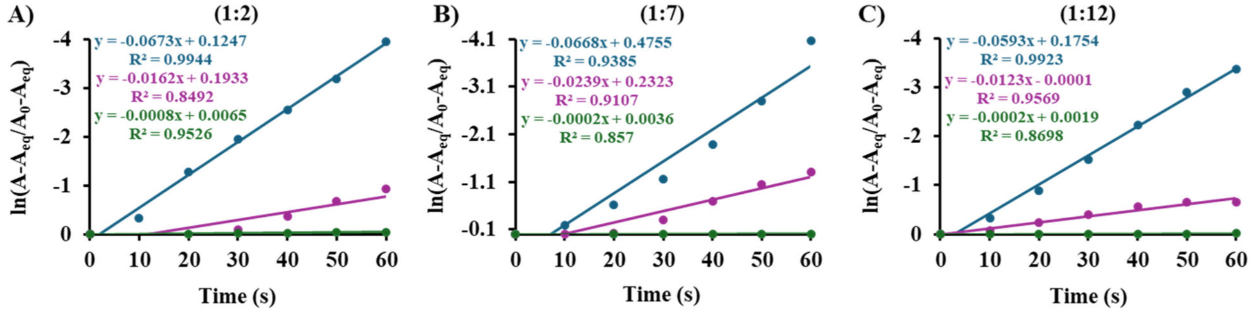

Figure S8. Pseudo first-order kinetic plots of PNP reduction with 2.0 (**green**), 4.0 (**purple**), and 8.0 (**blue**) μM [Au] for (**A**) 1:2, (**B**) 1:7, and (**C**) 1:12 molar ratios of Au:PEG, for preparations with GNPs-$PEG_{4.0}$.

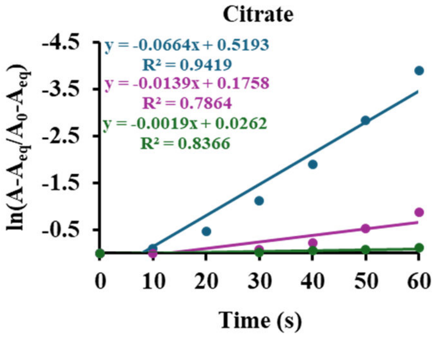

Figure S9. Pseudo first-order kinetic plots of PNP reduction with 2.0 (**green**), 4.0 (**purple**), and 8.0 (**blue**) μM [Au] for GNPs-citrate.

STABILITY DATA

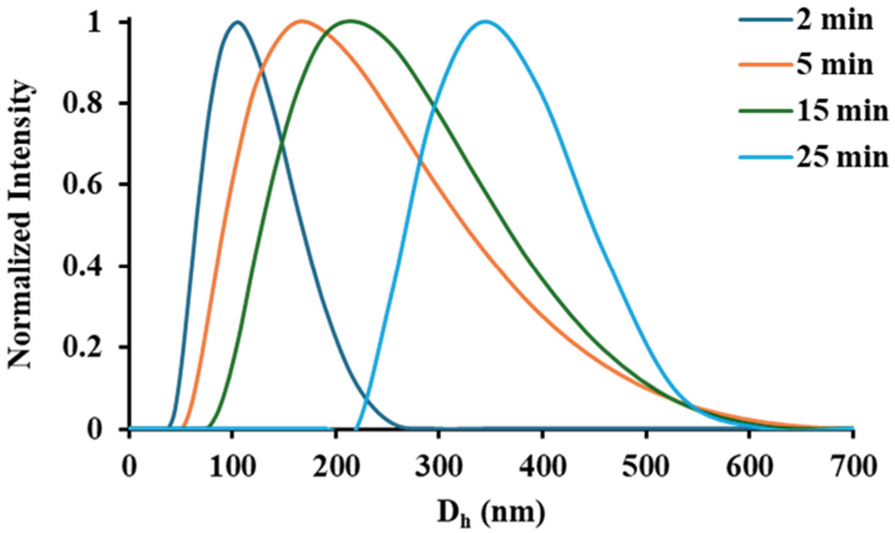

Figure S10. D_h of GNPs-PEG$_{6.0}$ (1:12), over time, in PNP reduction medium.
